# Estudio del efecto del tratamiento con fármacos antidiabéticos sobre el metabolismo óseo

**DOI:** 10.1515/almed-2024-0033

**Published:** 2024-03-25

**Authors:** Nuria Padilla Apuntate, Carmen G. Puerto Cabeza, Alba Gallego Royo, Nuria Goñi Ros, Claudia Abadía Molina, Javier Acha Pérez, Pilar Calmarza

**Affiliations:** Servicio de Bioquímica Clínica, 16488 Hospital Universitario Miguel Servet, Zaragoza, España; Servicio de Medicina Preventiva, 16488 Hospital Universitario Miguel Servet, Zaragoza, España; Servicio de Endocrinología y Nutrición, 16488 Hospital Universitario Miguel Servet, Zaragoza, España; Instituto de Investigación Sanitaria (IIS) Aragón, Zaragoza, España; Universidad de Zaragoza, Zaragoza, España; Centro de Investigación en Red de Enfermedades Cardiovasculares (CIBERCV), Instituto Salud Carlos III, Madrid, España

**Keywords:** diabetes mellitus tipo 2, marcadores remodelado óseo, fármacos antidiabéticos, pioglitazona

## Abstract

**Objetivos:**

La prevalencia de la diabetes mellitus tipo 2 (DMT2) está aumentando de forma exponencial en todo el mundo, habiéndose comprobado que estos pacientes tienen mayor riesgo de presentar fracturas óseas, con respecto a la población sana, por lo que resulta de gran relevancia el conocimiento del efecto de los fármacos antidiabéticos sobre el metabolismo óseo.

**Métodos:**

Estudio estadístico descriptivo, retrospectivo, de 106 pacientes en tratamiento con seis grupos de fármacos antidiabéticos: insulina, inhibidores de dipeptidilpeptidasa 4 (iDPP4), agonistas del receptor del péptido similar al glucagón tipo 1 (arGLP1), sulfonilureas, inhibidores del cotransportador de sodio-glucosa tipo 2 (iSGLT2) y pioglitazona, en los que se determinaron osteocalcina (OC), fosfatasa alcalina ósea (FAO) y telopéptido C-terminal del colágeno tipo 1 o beta-crosslaps (β-CTx).

**Resultados:**

Se encontraron concentraciones más elevadas de β-CTx en los pacientes tratados con pioglitazona que en los tratados con iDPP4 (p=0,035), iSGLT2 (p=0,020) y con arGLP1 (p<0,001), siendo los pacientes tratados con arGLP1 los que presentaron las concentraciones más bajas de β-CTx.

**Conclusiones:**

El tipo de tratamiento antidiabético recibido en pacientes que padecen DMT2 puede afectar el remodelado óseo. En nuestro estudio los pacientes que fueron tratados con pioglitazona mostraron las concentraciones más elevadas de β-CTx con respecto al resto de grupos de fármacos, lo cual parece indicar la conveniencia de evitar estos fármacos, sobre todo en mujeres postmenopáusicas con DMT2. Los fármacos arGLP1 presentaron los valores más bajos de β-CTx, por lo que podrían ejercer un efecto beneficioso sobre el metabolismo óseo.

## Introducción

La diabetes mellitus afecta a 422 millones de personas en todo el mundo y en los últimos años su prevalencia ha aumentado de forma exponencial, especialmente en países con ingresos medios y bajos [1]. Su forma más común es la diabetes mellitus tipo 2 (DMT2), la cual incluye a más del 90 % de los casos y se caracteriza por presentar resistencia a la insulina, a menudo acompañada de una deficiencia relativa de ésta [2].

Los pacientes con DMT2 presentan un mayor riesgo de fracturas óseas por fragilidad con respecto a la población sana [3] y al contrario de los casos de osteoporosis primaria, presentan valores de densidad mineral ósea (DMO) normales o incluso elevados, pero baja resistencia ósea. Esta baja resistencia ósea puede deberse a alteraciones en la microarquitectura y remodelado óseo, junto con una mineralización defectuosa, a la cual contribuyen también factores derivados de la enfermedad, como el aumento del estrés oxidativo y la inflamación [4].

El hueso es un tejido dinámico, sometido a un proceso continuo de remodelado, que se produce a lo largo de toda la vida e implica la resorción del tejido óseo antiguo y la formación de tejido óseo nuevo, con el fin de mantener la competencia biomecánica y la homeostasis mineral, previniendo así la aparición de lesiones. Cuando se produce un enlentecimiento de este proceso puede originarse un tejido óseo con densidad normal o alta, pero con un elevado riesgo de fractura, como en el caso de los pacientes con DMT2, en los cuales parece existir una disminución en el recambio óseo [5] debida sobre todo a los efectos directos o indirectos de la hiperglucemia, la hiperinsulinemia de las primeras fases de la enfermedad, la obesidad y el efecto de los fármacos antidiabéticos orales.

En este sentido, algunos estudios histomorfométricos han permitido comprobar que en los pacientes con DMT2 existe una reducción en el número de osteoblastos [6] y en un metaanálisis, que incluía 22 estudios de pacientes con DMT2, encontraron que en estos pacientes existía una disminución en el remodelado óseo, con concentraciones bajas de osteocalcina (OC), marcador de formación ósea, así como de beta crosslaps (β-CTx) (resorción ósea) en relación con los controles sanos [7]. Estos resultados fueron corroborados también por otro metaanálisis más reciente [8].

Sin embargo, es importante resaltar que, aunque disponemos de métodos adecuados para la determinación en suero de los marcadores de remodelado óseo (MRO) [9] y a pesar del potencial papel que puede ejercer la disminución del recambio óseo en el aumento de riesgo de fracturas por fragilidad en los pacientes con DMT2, no se tiene todavía evidencia de que los marcadores de recambio óseo puedan predecir la aparición de fracturas en estos pacientes [5] y que tanto la medida de la densidad mineral ósea (determinación del T-Score) como el *Fracture Risk Assessment Tool* (FRAX) subestiman la predicción de fracturas en pacientes con diabetes, especialmente en los pacientes con DMT2.

Asimismo, algunos estudios realizados en las dos últimas décadas ponen de manifiesto el impacto del tratamiento con fármacos antidiabéticos en la densidad mineral ósea [10, 11], pudiendo contribuir a la alteración del remodelado óseo en los pacientes con DMT2 y al aumento del riesgo de fractura ósea en una población, ya vulnerable. Estas fracturas por fragilidad que afectan a los pacientes diabéticos pueden ser muy graves, llegando a producir discapacidad o incluso la muerte.

El objetivo de este estudio ha sido determinar el efecto de los fármacos utilizados en el tratamiento de la DMT2 en el metabolismo óseo de estos pacientes mediante la determinación de la concentración sérica de los MRO de formación (OC y FAO) y de resorción ósea (β-CTx), en una muestra poblacional adscrita a nuestro sector sanitario.

## Materiales y métodos

Estudio descriptivo y retrospectivo de 106 pacientes (36,8 % mujeres, todas ellas postmenopáusicas), con una edad media de 67±8 años, afectos de DMT2 y en tratamiento con fármacos antidiabéticos, a los que se les solicitó un análisis de sangre que incluía la determinación de los MRO, en el periodo de tiempo comprendido entre mayo de 2020 y febrero de 2021. La población de estudio fue seleccionada en base a edades y proporción de hombres y mujeres similares y se sesubdividió en seis grupos según la estrategia de tratamiento antidiabético recibida. A los pacientes de todos los grupos se les administraba metformina.

Los grupos establecidos, según tratamiento, fueron los siguientes:

– Grupo 1: Insulina: 15 pacientes, edad mediana (EM): 68 años (Q1–Q3: 61–78 años).
– Grupo 2: iDPP4: 28 pacientes, EM: 68 años (Q1–Q3: 66–72 años).
– Grupo 3: arGLP1: 16 pacientes, EM: 64 años (Q1–Q3: 58–73 años).
– Grupo 4: Sulfonilureas: 16 pacientes, EM: 66 años (Q1–Q3: 58–71 años).
– Grupo 5: iSGLT2: 15 pacientes, EM: 68 años (Q1–Q3: 59–70 años).
– Grupo 6: Tiazolidindionas (pioglitazona): 16 pacientes, EM: 68 años (Q1–Q3: 62–73 años).

Se excluyeron los pacientes que presentaran otras patologías o que estuvieran en tratamiento con fármacos, que pudiesen interferir en el metabolismo óseo. Ninguno de los pacientes tomaba suplementos de vitamina D y el tiempo mínimo del tratamiento indicado en cada uno de los pacientes era igual o superior a tres años.

Este estudio cumple con todas las regulaciones nacionales, políticas institucionales y principios éticos de la Declaración de Helsinki y fue aprobado por el Comité de Ética de Investigación de la Comunidad Autónoma de Aragón (CEICA).

La extracción de las muestras de sangre se realizó por la mañana tras un ayuno mínimo de **ocho** horas e inmediatamente después de la obtención del suero, fueron almacenadas a −80 °C para ser analizadas en una misma tanda. No se administró ninguna pauta dietética específica, habiendo seguido cada paciente su hábito dietético habitual.

En cada uno de los pacientes se determinó la concentración de los siguientes parámetros: OC, FAO y β-CTx. Los marcadores OC y β-CTx se cuantificaron mediante enzimoinmunoanálisis (N-MID Osteocalcin ELISA, Inmunodiagnostic System Ltd, Boldon, UK y Serum Crosslaps, Inmunodiagnostic Systems Ltd, Boldon, UK), respectivamente, al igual que FAO (Microvue BAP, EIA, Quidel corporation, San Diego, CA, EEUU). Para cada uno de los parámetros se emplearon los siguientes rangos de referencia: β-CTx (115–684 pg/mL), OC (5,8–39,8 ng/mL) y FAO (12–43 UI/L). El umbral de detección para OC, FAO y β-CTx fue de 0,5 ng/mL, 0,7 U/L y 0,020 ng/mL, respectivamente, y los coeficientes de variación intraensayo no superaron 2,2 ng/mL, 5,8 U/L y 3 ng/mL, respectivamente. Los coeficientes de variación interensayo no superaron 5,1 ng/mL, 7,6 U/L y 10,9 ng/mL, respectivamente, para cada uno de los parámetros indicados.

Para el análisis estadístico se empleó el programa IBM SPSS^®^ Statistics 21.0 (IBM Corporation, Armonk, NY). Para la comprobación de la normalidad global de todos los parámetros estudiados, empleamos la prueba de normalidad de Kolmorogov Smirnoff, obteniendo como resultado que edad, peso y talla siguen una distribución normal, mientras que el índice de masa corporal (IMC), edad de inicio de la diabetes mellitus, hemoglobina glicada (HbA_1c_), OC, FAO y β-CTx, no siguen una distribución normal. Para comprobar la normalidad de estos parámetros según el grupo de fármacos empleado se utilizó el test de Shapiro–Wilk (tamaño muestral por grupos <50). Se observó que todos estos parámetros no cumplían condiciones de normalidad en alguno de los grupos de fármacos administrados, por lo que se han empleado pruebas no paramétricas para su estudio.

Para comprobar si existían diferencias en cada uno de los parámetros citados en los distintos grupos de pacientes, según tratamiento, se empleó el test H de Kruskall Wallis, completándose el análisis con el test de Bonferroni para comparaciones múltiples entre categorías, en aquellas variables en las que se obtuvieron diferencias significativas. Se estableció un nivel de significación de p <0,05. Para determinar si existían diferencias entre los distintos grupos respecto al sexo se empleó la prueba de Chi al cuadrado y para determinar si existía correlación entre las distintas variables cuantitativas se utilizó el test Rho de Spearman.

## Resultados

Los datos antropométricos de los pacientes, así como los años de evolución de la DMT2, el control glucémico (HbA_1c_) y el tiempo transcurrido desde el inicio de la DMT2, en los distintos grupos de pacientes, se muestran en la Tabla 1. No se encontraron diferencias estadísticamente significativas entre los diferentes tipos de tratamiento en cuanto a sexo (p=0,956). Tampoco se encontraron diferencias en relación a la edad (p=0,193), así como en la concentración de HbA_1c_ (p=0,218). Sin embargo, sí que existían diferencias entre los distintos grupos para las variables peso, IMC y edad al inicio de la DMT2 (p=0,002, p=0,001 y p=0,023 respectivamente). Concretamente, en el peso se observaron diferencias significativas entre el grupo iDPP4 vs. arGLP1 (p=0,003) y entre el grupo de pacientes tratados con pioglitazona vs. arGLP1 (p=0,007). En cuanto al IMC, se observaron estas mismas diferencias entre el grupo iDPP4 vs. arGLP1 (p=0,001) y entre el grupo pioglitazona vs. arGLP1 (p=0,025), encontrando en ambos casos valores más elevados en los pacientes tratados con arGLP1.

**Tabla 1:** Datos antropométricos de los pacientes, años inicio diabetes mellitus tipo 2 y HbA_1c_, según tratamiento antidiabético recibido.

Tratamiento antidiabético	Sexo mujer, %	Edad, años^a^	Peso, kg^a^	Altura, cm^a^	IMC, kg/m^2a^	Años inicio DMT2^a^	HbA_1c_, %^a^
Insulina, n=15	46,7	68 (61–78)	77,6 (72,2–83,0)	161 (150–167)	29,8 (26,6–33,6)	13,0 (11,0–16,0)	8,10 (7,40–8,85)
iDPP4, n=28	35,7	68 (66–72)	73,2 (71,0–78,9)	167 (158–171)	25,9 (24,9–29,5)	9,5 (8,0–11,0)	7,15 (6,50–7,50)
arGLP1, n=16	33,3	64 (58–73)	96,5 (85,0–99,8)	164,5 (158–171)	34,4 (31,2–35,4)	10,0 (7,5–11,0)	7,40 (6,95–8,10)
Sulfonilureas, n=16	37,5	66 (58–71)	74,9 (66,9–90,0)	165 (158–167)	28,7 (21,3–26,1)	10,5 (10–16,3)	7,15 (6.65–7,90)
iSGLT2, n=15	31,3	68 (59–70)	83,6 (77,0–92,3)	166 (159–171)	30,0 (27,9–32,7)	11,5 (8,8–13,3)	7,30 (7,00–7,92)
Pioglitazona, n=16	40	68,5 (62–73)	73,5 (63,9–79,8)	160 (153–164)	27,1 (21,6–27,1)	11,0 (10,0–14,0)	7,30 (6,65–8,00)

^a^Resultados expresados como mediana y rango intercuartílico. IMC, índice de masa corporal; DMT2, diabetes mellitus tipo 2; HbA_1c_, hemoglobina glicada; iDPP4, inhibidores de dipeptidilpeptidasa; arGLP1, agonistas del receptor del péptido similar al glucagón tipo 1; iSGLT2, inhibidores del cotransportador de sodio-glucosa tipo 2.

Para la variable edad al inicio de la diabetes, se observaron diferencias estadísticamente significativas en la comparación entre el grupo iDPP4 vs. insulina (p=0,025), siendo superior en el grupo de pacientes tratados con insulina. Todos los pacientes presentaban una función hepática y renal normal y llevaban un mínimo de tres años recibiendo el tratamiento asignado.

**Figura 1:**
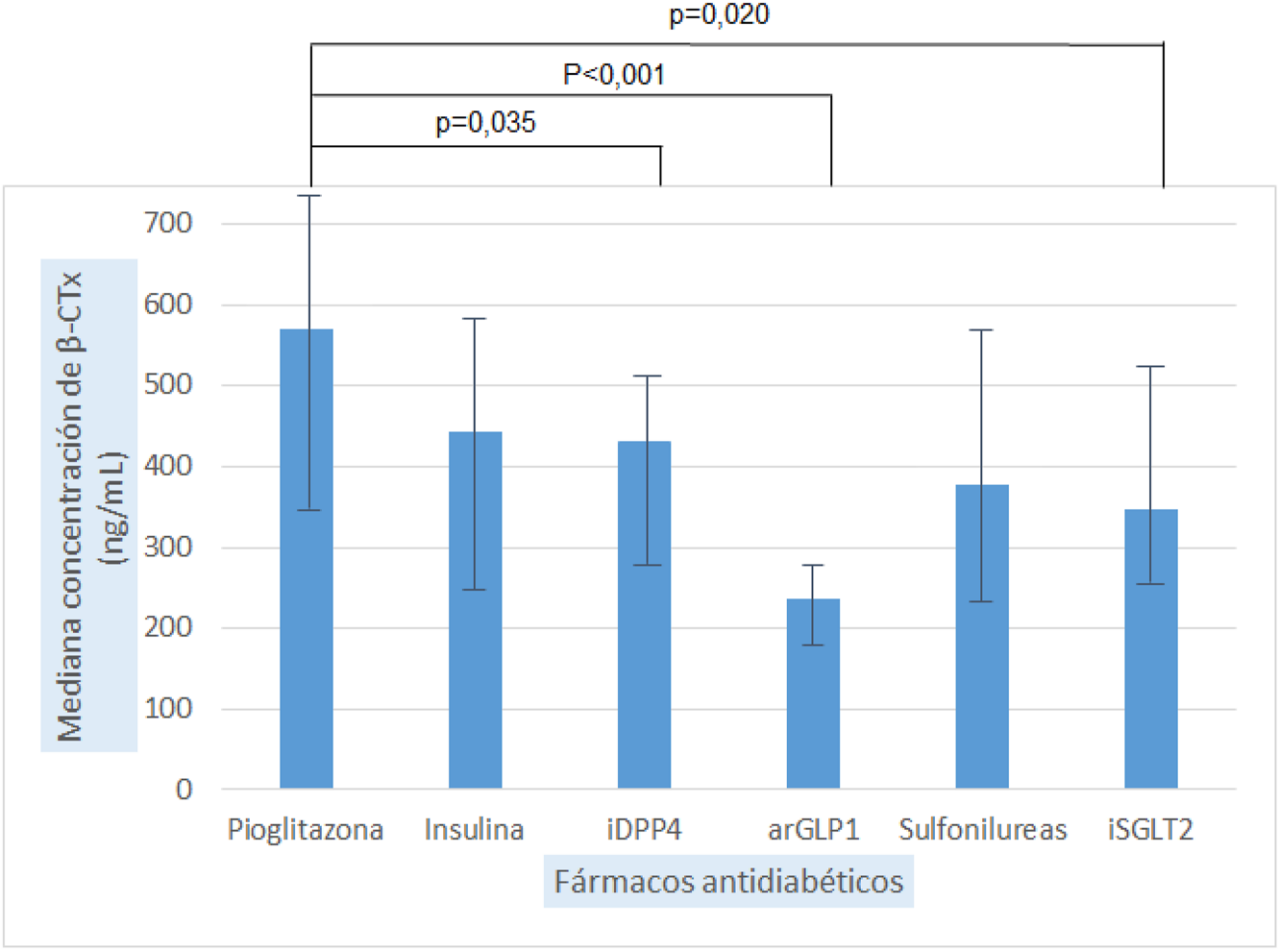
Diferencias en la concentración de los marcadores de remodelado óseo (β-CTx), según el tratamiento antidiabético recibido. Las barras indican mediana y rango intercuartílico. iDPP4, inhibidores de dipeptidilpeptidasa 4; arGLP1, agonistas del receptor del péptido similar al glucagón tipo 1; iSGLT2, inhibidores del cotransportador de sodio-glucosa tipo 2.

Al comparar si existían diferencias significativas en la concentración de los marcadores óseos, según la prescripción de los distintos grupos de fármacos, se encontraron diferencias para los parámetros: OC (p=0,026), FAO (p=0,007) y β-CTx (p=0,002), según se muestra en la Tabla 2. Tras la aplicación del test de Bonferroni solamente se mantuvieron estas diferencias para β-CTx, el cual se encontraba más elevado en los enfermos que recibieron pioglitazona, al compararlo con los que fueron tratados con iSGLT2 (p=0,020), iDPP4 (p=0,035) y arGLP1 (p<0,001), según se muestra en la Figura 1.

**Tabla 2:** Concentración de los marcadores de remodelado óseo, según tratamiento antidiabético recibido.

Tratamiento antidiabético	Osteocalcina^a^, ng/mL	Fosfatasa alcalina ósea^a^, ng/mL	β-Crosslaps^a^, pg/mL
Insulina	8,2 (5,2–16,6)	31 (24,6–36,4)	456,6 (244,5–589,6)
iDPP4	5,9 (5,4–7,7)	28,5 (23,6–36,7)	436,2 (269,1–508,9)
arGLP1	6 (3,2–8,7)	22 (17,8–27,1)	239,8 (196,4–279,3)
Sulfonilureas	10 (5,8–12,5)	21,6 (18,4–26,8)	379,8 (232,8–565,8)
iSGLT2	5,4 (3,9–10,6)	22,4 (18,6–24,8)	349,1 (249,6–515,8)
Pioglitazona	10,6 (6,9–15,4)	23,3 (19,5–27,9)	572,9 (344,2–718)

^a^Resultados expresados como mediana y rango intercuartílico. iDPP4, inhibidores de dipeptidilpeptidasa; arGLP1, agonistas del receptor del péptido similar al glucagón tipo 1; iSGLT2, inhibidores del cotransportador de sodio-glucosa tipo 2.

El tratamiento con insulina, junto con el tratamiento con sulfonilureas y pioglitazona, mostraron las concentraciones más elevadas de los marcadores de formación ósea.

En el caso de los iDPP4, se observaron concentraciones elevadas de FAO, en comparación con las observadas en el tratamiento con otros fármacos, pero no ocurrió lo mismo con la concentración de OC, encontrándose la concentración de la misma entre las más bajas, al compararla con la encontrada en el tratamiento con el resto de fármacos.

En cuanto a los marcadores de resorción ósea, se obtuvieron las concentraciones más bajas de β-CTx en el tratamiento con arGLP1, seguidos de los obtenidos con el tratamiento con isGLT2 y sulfonilureas. El tratamiento con pioglitazona fue el que mostró los resultados más elevados de β-CTx. Todos estos resultados se encuentran representados en la Tabla 2.

Hemos encontrado que existe correlación positiva significativa entre OC y FAO (Rho Spearman 0,346, p=0,001), entre OC y β-CTx (Rho Spearman 0,552 p<0,001) y entre FAO y β-CTx (Rho de Spearman 0,298 p=0,003). Cuando estudiamos la correlación existente entre los distintos marcadores óseos según el tipo de tratamiento farmacológico recibido, encontramos una correlación positiva significativa entre OC y β-CTx (Rho Spearman 0,604, p=0,017) en el grupo de pacientes tratados con insulina. Además, en el grupo de pacientes tratados con iDPP4 hallamos una correlación positiva significativa entre β-CTx y FAO (Rho Spearman 0,476, p=0,064).

En el grupo de tratamiento con iSGLT2 se observó correlación positiva significativa entre β-CTx y OC (Rho Spearman 0,656, p=0,006) y en el grupo de tratamiento con pioglitazona correlación positiva significativa entre FAO y OC (Rho Spearman 0,531, p=0,042), así como entre β-CTx y OC (Rho Spearman 0,531, p=0,042). En los grupos de tratamiento con arGLP1 y con sulfonilureas no se observó correlación significativa alguna entre los distintos marcadores óseos.

## Discusión

Nuestros resultados permiten establecer una asociación del fármaco arGLP1 con la menor tasa de recambio óseo, presentando las concentraciones más bajas de MRO de formación y de resorción. Por el contrario, los fármacos insulina y pioglitazona se asocian a una tasa de recambio óseo mucho más elevada, presentando los valores más altos de ambos tipos de MRO.

Los resultados encontrados en el grupo de pacientes tratados con insulina, son concordantes con los obtenidos por algunos investigadores [12, 13], los cuales sugieren que la insulina ejerce un potente efecto anabólico sobre los osteoblastos. Sin embargo, Ivaska y cols [14]. sostienen la idea de que la insulina disminuye la concentración de β-CTx y de OC, en contraposición con nuestros resultados.

Por otra parte, Kanazawa y cols [15] observaron que la terapia con insulina se asociaba con mayor riesgo de fracturas. Además, Napoli y cols [16] e Hidayat y cols [17] posteriormente comprobaron que puede ser debido a que produce un aumento en la frecuencia de eventos hipoglucémicos severos y por tanto un mayor riesgo de caídas.

Los fármacos antidiabéticos iDPP4 y arGLP1 se considera que tienen un efecto positivo sobre el metabolismo óseo, incrementando la función osteoblástica y disminuyendo la función osteoclástica, aunque existen algunos resultados discrepantes como los encontrados por Yang y cols [18], los cuales señalan que el aumento de la actividad de iDPP4 puede promover indirectamente la resorción ósea e inhibir la formación ósea. Sin embargo, en un estudio reciente llevado a cabo en ratones en los que se había simulado DMT2 [19] se encontraron valores disminuidos de OC y aumentados de TRAP 5b, los cuales revertían tras la administración de linagliptina (iDPP4). Abdi y cols [20] utilizando dos modelos de ratas Wistar con DMT2 tratadas con fármacos antidiabéticos de tipo iDPP4 y arGLP1, respectivamente, llegaron a la conclusión de que ambos tipos de fármacos disminuyen la resorción ósea, obteniendo un perfil óseo algo más favorable en el grupo de ratas tratadas con arGLP1. Nuestros resultados apuntan en esta misma dirección, obteniéndose la menor concentración de los marcadores de resorción ósea en el grupo de pacientes tratados con arGLP1.

Respecto al grupo de fármacos iSGLT2, Dong y cols [21] encontraron que canagliflozina aumenta tanto los marcadores de resorción (β-CTx) como los marcadores de formación ósea (OC); sin embargo, aunque dapagliflozina y empagliflozina (también fármacos iSGLT2) no mostraron ningún efecto significativo sobre los marcadores de formación ósea, dapagliflozina aumentaba significativamente la resorción ósea, por lo que consideran que presenta un efecto negativo sobre el contenido óseo. Por otro lado, en la revisión llevada a cabo por Jackuliak y cols [22] se sugiere que el grupo de antidiabéticos iSGLT2 presenta propiedades neutras para los marcadores, tanto de resorción como de formación ósea y en el metaanálisis llevado a cabo por Tang y cols [23] tampoco se encontró mayor riesgo de fractura en los pacientes tratados con iSGLT2. En nuestro estudio los pacientes tratados con iSGLT2 presentaron valores intermedios tanto para OC como para β-CTx.

En cuanto al grupo de pacientes tratados con sulfonilureas, en la revisión de Jackuliak y cols [22] se afirma que el riesgo de fractura en estos pacientes es similar al de otros grupos, pero destacan un aumento en la formación y una disminución en la resorción ósea. Nuestro estudio muestra resultados similares, puesto que la concentración de OC está por encima de la mediana de los distintos grupos de fármacos y la de β-CTx por debajo.

En nuestro estudio, el grupo de pacientes tratados con piogliotazona destaca por presentar las concentraciones más elevadas de los marcadores de resorción ósea, en relación al resto de fármacos estudiados. De hecho, solo se mantienen las diferencias encontradas inicialmente en el caso de β-CTx, con concentraciones más elevadas en los enfermos tratados con pioglitazona, al compararlos con los que recibieron iSGLT2, iDPP4 y arGLP1, siendo las diferencias más acusadas entre los tratados con pioglitazona y los tratados con arGLP1.

En este sentido, algunos autores parecen coincidir en que dicho fármaco aumenta la resorción ósea, aunque existen algunas discrepancias. Así, mientras que Mori y cols [24] encontraron que la pioglitazona aumentaba la resorción ósea, independientemente de la edad y del género, Xiao y cols [25] afirman que la pioglitazona parece actuar inhibiendo la formación de hueso, sin afectar a la resorción ósea.

Los resultados encontrados en nuestro estudio sugieren la influencia de la administración de estos fármacos en el proceso de resorción ósea, presentando los pacientes tratados con pioglitazona un aumento estadísticamente significativo en la concentración de los MRO de resorción, respecto a los pacientes tratados con otro tipo de fármacos antidiabéticos. Esto podría indicar la necesidad de plantearse evitar estos fármacos, sobre todo en mujeres postmenopáusicas con DMT2.

Sin embargo, los fármacos arGLP1 presentan los valores más bajos en los MRO de resorción ósea, por lo que podrían ejercer un efecto beneficioso sobre el metabolismo óseo de los pacientes tratados con este tipo de fármacos.

Como conclusión, podemos decir que el tipo de tratamiento antidiabético recibido puede afectar al remodelado óseo y por lo tanto a la salud ósea de los pacientes con DMT2.
